# A Natural Moisture Gradient Affects Soil Fungal Communities on the South Shore of Hulun Lake, Inner Mongolia, China

**DOI:** 10.3390/jof9050549

**Published:** 2023-05-10

**Authors:** Xin Chen, Yujue Wang, Yao Wang, Yushu Zhang, Yuting Shen, Xiaojia He, Chunwang Xiao

**Affiliations:** 1 College of Life and Environmental Sciences, Minzu University of China, Beijing 100081, China; 2 Beijing Key Laboratory of Ecological Function Assessment and Regulation Technology of Green Space, Beijing Academy of Forestry and Landscape Architecture, Beijing 100102, China; 3 The Administrative Center for China’s Agenda 21, Beijing 100038, China

**Keywords:** south shore of Hulun Lake, natural moisture gradient, soil fungal community, community diversity and structure, environmental factors

## Abstract

Soil moisture content (SWC) can change the diversity and composition of soil fungal communities by affecting soil texture and soil nutrients. To explore the response of soil fungal communities to moisture in the grassland ecosystem on the south shore of Hulun Lake, we set up a natural moisture gradient that was subdivided into high (HW), medium (MW), and low (LW) water contents. Vegetation was investigated by quadrat method, and aboveground biomass was collected by the mowing method. Soil physicochemical properties were obtained by internal experiments. The composition of the soil fungal community was determined using high-throughput sequencing technology. The results showed significant differences in soil texture, nutrients, and fungal species diversity under the moisture gradients. Although there was significant clustering of fungal communities in different treatments, the fungal community composition was not significantly different. According to the phylogenetic tree, the Ascomycota and Basidiomycota were the most important branches. The fungal species diversity was smaller when SWC was higher, and in this environment (HW), the fungal-dominant species were significantly related to SWC and soil nutrients. At this time, soil clay formed a protective barrier for the survival of the dominant classes Sordariomycetes and Dothideomycetes and increased their relative abundance. In summary, the fungal community responded significantly to SWC on the southern shore of the Hulun Lake ecosystem in Inner Mongolia, China, and the fungal community composition of the HW group was stable and easier to survive.

## 1. Introduction

Soil microorganisms are the most active components of soil ecosystems and are involved in many key soil processes, such as organic matter formation and transformation, and elemental biogeochemical cycling [1,2]. In a global change context, soil microbial community dynamics are important aspects in the process of response to terrestrial ecosystems and adaptation to global changes [3,4]. Fungi are critical members of the soil microbial composition, and although numerically less than bacteria, they play a significant role [5], and are major decomposers of refractory organic matter [6]. They not only contribute to soil structure regulation and soil formation [7], but also are more sensitive to changes in soil physicochemical properties and can be used as indicators of soil quality [8]. Meanwhile, soil fungi can form symbiosis with plants and drive soil productivity changes together with plants [9]. Soil decomposing fungi can also enhance plant productivity by producing a series of extracellular enzymes and decompose organic matter in the ecosystem to promote nutrient cycling [10]. Furthermore, fungi can help vegetation to absorb water from the soil, alleviate water stress in plants [11], and improve the disease resistance of the root system [12]. Additionally, it has been pointed out that soil fungi have good resistance and show strong survival in harsh environments [13]. Therefore, clarifying soil fungal community composition and diversity can provide a scientific basis for evaluating soil quality and fertility.

Soil water content (SWC) is one of the important factors that maintains soil microbial activity and affects ecosystem function [14]. Directly controlling the diffusion and transport of soil nutrients, it also affects the diversity of soil microorganisms [15]. Physicochemical properties such as SWC, pH, soil total nitrogen (TN), soil organic carbon (SOC), and soil texture are important factors influencing fungal community diversity and driving fungal community composition [16,17,18,19]. Different microflora has different response characteristics to changes in SWC, and SWC directly or indirectly affects microbial community composition [20]. Lu et al. [21] found that wetland water accumulation variations and seasonal shifts in wet and dry conditions can lead to changes in soil fungal abundance and diversity. Bacterial communities tend to be more affected than fungal communities are under drought conditions, and fungal communities in wet soils are more sensitive to changes in water content [22]. Moreover, soil organic matter affects the microbial abundance [23] and decomposition rate [24], and the above processes can be determined by soil moisture [25]. In conclusion, the effects of moisture condition changes on both soil processes and ecosystem functions can depend on soil microbial community structure [26]. It is important to study the response of soil microbial communities and changes in ecosystem function to different moisture conditions.

Hulun Lake is a large lake in the high-latitude, semi-arid region of northern China, a unique natural lake ecosystem with diversity and ecological functions similar to those of arid regions [27]. However, due to the continuous droughts in spring and summer and the increased frequency of snowstorms in winter in the Hulun Lake basin, the climate of the region tends to be warm and dry, the water recharge of Hulun Lake is insufficient, and the lake surface is shrinking; thus, the ecological environment is becoming increasingly serious [28]. Currently, research on the response of Hulun Lake basin to climate change has become a hot topic [29], but most research has focused on the impact of climate change on the ecology of Hulun Lake wetlands [30]. Few studies have been conducted on soil microorganisms in grassland ecosystems under soil moisture changes on the south shore of the lake. In this paper, a natural moisture gradient from the lake shore toward the inland was established for exploring the effects of soil moisture on soil fungal communities in the grassland ecosystem of the south shore of Hulun Lake. We hypothesize that (1) soil physicochemical properties appear differently in the grassland ecosystem of the south shore of Hulun Lake during moisture changes; (2) soil fungal community diversity and structural composition of the grassland ecosystem on the south shore of Hulun Lake show regular changes with changes in SWC; and (3) SWC can affect soil texture and aeration, and water, as a transport media, can transport organic matter everywhere and improve soil nutrient availability, thus affecting the microbial environment. Therefore, we hypothesize that SWC and soil organic matter can influence soil fungal community composition on the south shore meadow ecosystem of Hulun Lake.

## 2. Materials and Methods

### 2.1. Study Site

Hulun Lake is the largest lake in the Inner Mongolia Autonomous Region, located at 117°00′10″–117°41′40″ E and 48°30′40″–49°20′40″ N. The lake surface is irregularly rectangular. This study was carried out in the grassland ecosystem of the south shore of Hulun Lake, characterized by low anthropogenic disturbance and single grazing. The area is a temperate grassland, and the establishment species are mainly Stipa krylovii Roshev and Leymus chinensis (Trin.) Tzvel. The climate is characterized by a semi-arid continental climate, with an annual precipitation of about 280 mm and an average annual evaporation of 5–6 times the annual precipitation. In August 2019, we established a natural moisture gradient sample strip on the south shore of Hulun Lake, extending about 25 km landward along the lake, with a total of nine sampling sites, and three 10 m × 10 m replicate plots were randomly established in each sample site. Using a handheld GPS, each sample site was located at a distance of about 2–5 km. The sample site closest to the lake was sample point 1, and each sample point was further away from the lake in increasing order. We divided the natural moisture gradient sample strip into different groups of high water content (HW), medium water content (MW), and low water content (LW) based on distance from the lake and SWC (Table 1, Figure S1).

### 2.2. Field Sampling and Lab Analysis

We randomly selected three 1 m × 1 m quadrats in each sample plot, and the aboveground biomass (AGB) within each quadrat was mowed, and brought back to the laboratory to dry to a constant weight in a desiccator, and weighed. Using a soil auger boring of 8 cm in diameter and 10 cm in height, a 0–10 cm soil layer was drilled in each of the mowed sample quadrat, three soil samples were mixed into a soil sample in each plot, three soil samples were collected for each site, and a total of 27 soil samples were collected in the natural moisture gradient. The soil was sieved at 2 mm to remove obvious roots. The sieved fresh soil samples were divided into two parts, and one part was stored at 4 °C for the determination of basic physical and chemical properties of the soil. Soil bulk density (BD) was measured by the cutting ring method and SWC was measured by the drying method. Soil pH was measured using a combination glass electrode. SOC and TN were measured with the elemental analyzer FLASH 2000. Soil texture (sand, clay, and silt content) was analyzed with a laser particle analyzer Mastersizer 2000. The other part of fresh soil samples was put into sterile centrifuge tubes and stored in a refrigerator at −80℃ and sent to the Research Center for Eco-Environment Sciences, Chinese Academy of Sciences for high-throughput sequencing of soil microorganisms.

### 2.3. DNA Extraction, Polymerase Chain Reaction (PCR) Amplification, Sequencing Analysis and ASV Generation

Genomic DNA was extracted from 0.5 g of fresh soil using a PowerSoil DNA Isolation Kit (MO BIO). DNA quality was determined using a NanoDrop spectrophotometer (Nano-100, Aosheng Instrument Co Ltd., Suzhou, China). For fungi, PCR was performed to amplify the ITS2 region using primers ITS3 (5′-GCATCGGAAGAACGCAGC-3′) and ITS4 (5′-TCCTCCGCTTATTGATATGC-3′) [31].The amplification conditions were as follows: 95 °C for 5 min, 40 cycles of 94 °C for 30 s, annealing at 53 °C for 30 s, extension at 72 °C for 40 s, and a final extension at 72 °C for 8 min. PCR products were extracted on a 2% agarose gel using the GENEray DNA Gel Extraction Kit for further purification and quantification using a NanoDrop spectrophotometer. The purified amplicons were sequenced by Illumina HiSeq 2500 at the Research Center for Ecological and Environmental Sciences, Chinese Academy of Sciences to determine the microbial community structure of the samples.

ITS gene sequences were processed with USEARCH10 [32]. First, paired reads were merged, then primers and quality filters were removed. In addition, single-base precision ZOTUs (zero-radius OTU) were obtained by UNOISE3 denoising to predict biological sequences and filter chimeras. The sequences named amplicon sequence variant (ASV) were modified to facilitate identification. Subsequently, the utax_reference_dataset_all_04.02.2020.fasta.gz from the UNITE database (http://unite.ut.ee, 27 May 2020) was used with USEARCH10 [33,34] for fungal sequence identification, followed by taxonomic annotation. Finally, the sequences of specific ASVs were obtained and the ASV table was obtained by USEARCH10.

### 2.4. Data Analysis

The AGB, soil physicochemical properties, and relative abundance of fungi under different soil water contents were estimated by one-way ANOVA with SPSS 26 and Duncan’s post-hoc test. The “hclust” package was used in R to group the raw data after hierarchical clustering. Soil microbial diversity was computed using the “Vegan” package in R 3.6.0 software. Box line plots of fungal alpha diversity and stacked histograms of species composition were plotted using the “Hmisc” and “ggplot2” packages. Fungal β-diversity was analyzed in principal coordinates using the “Euclidean” distance matrix and visualized through the ImageGP website. The fungal-specific ASVs and shared ASVs sequences in different soil water contents were visualized using the “VennDiagram” package in R to obtain Venn diagrams. The sequence alignment was performed using Muscle software, followed by the rapid construction of maximum likelihood method (ML) evolutionary trees using IQ-TREE, and the obtained evolutionary tree data were imported into http://itol.embl.de/ (2 November 2022) for beautification. The Spearman correlation was calculated using the "psych" package in R, and corrected for p-values using the “fdr” method, followed by a correlation heat map of environmental factors and fungal strains using “pheatmap”.

## 3. Results

### 3.1. Environmental Factors under a Natural Moisture Gradient

SWC, soil clay, and sand content, SOC and TN were significantly different in the different moisture gradients. Among them, SOC and TN showed a trend of first increasing and then decreasing with the decrease in SWC (Table 2). Aboveground biomass, as a biological factor, also had a trend of variability under the moisture gradient (Table 2).

### 3.2. Phylogenetic Distribution of the Dominant Fungal Taxa under a Natural Moisture Gradient

**Figure 1:**
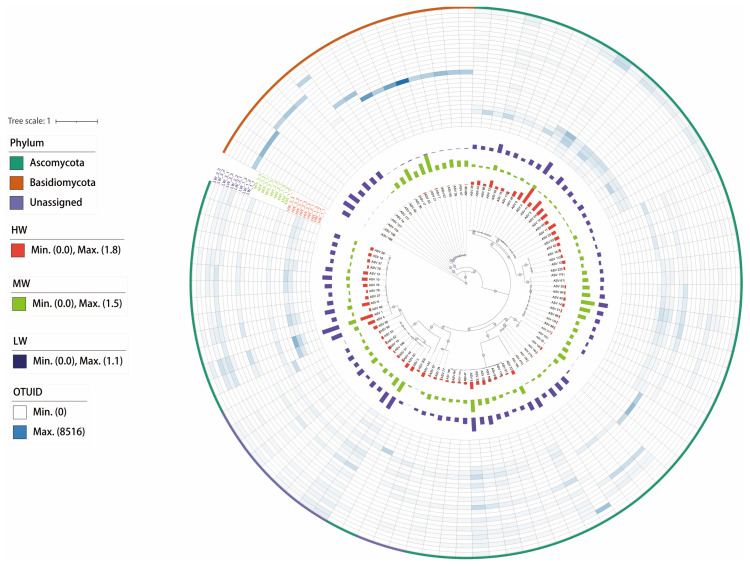
The phylogenetic tree shows the basic situation of fungal communities under a natural moisture gradient on the south shore grassland ecosystem of Hulun Lake. HW: high water content; MW: medium water content; LW: low water content. The ring colors represent fungal clades; the bars represent the relative abundance of ASVs; the heat map represents the correlation between ASVs, and the tree represents the results of hierarchical clustering.

The fungi were divided into three clades, and Ascomycota and Basidiomycota were the dominant clades (Figure 1). The ASVs in the Basidiomycota developmental clade were more strongly correlated with each other. At the near roots, the Basidiomycota and Unassigned clades clustered together and at the far roots, the Basidiomycota and Unassigned clades gradually separated, indicating that the two were more closely related at the beginning of development. The relative abundance of microorganisms under MW and LW was higher in the overall phylogeny (Figure 1).

### 3.3. Soil Fungal Relative Abundance and Diversity under a Natural Moisture Gradient

Figure S2 shows that the rarefaction curves of soil samples under a natural moisture gradient tends to be smooth, indicating that the sequencing tended to be saturated and the sampling was reasonable.

**Figure 2:**
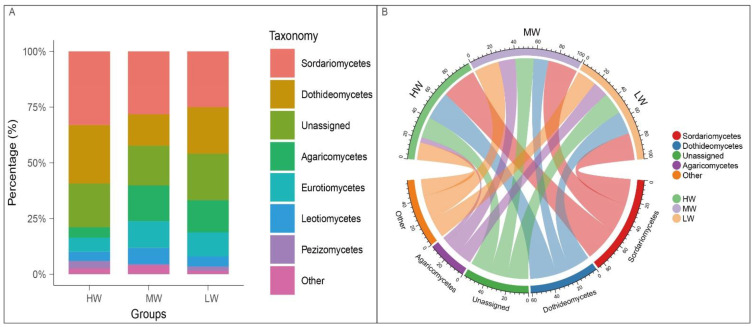
The relative abundances under a natural moisture gradient on the south shore grassland ecosystem of Hulun Lake are represented by stacked column diagram (**A**) and chord diagram (**B**), respectively. HW: high water content; MW: medium water content; LW: low water content.

There was no significant difference between the dominant phyla Ascomycota and Basidiomycota (Table 3). At the fungal class level, most sequences belonged to Sordariomycetes, Dothideomycetes, Agaricomycetes, etc. (Figure 2A). It showed a different cross-biotic community distribution and the relative abundance was Sordariomycetes > Dothideomycetes > Agaricomycetes (Figure 2B). Among them, there were intergroup differences in the dominant species Dothideomycetes. (Table 4). In addition, the relative abundance of the major fungal phyla varied in trend under a natural moisture gradient (Figure S3).

**Figure 3:**
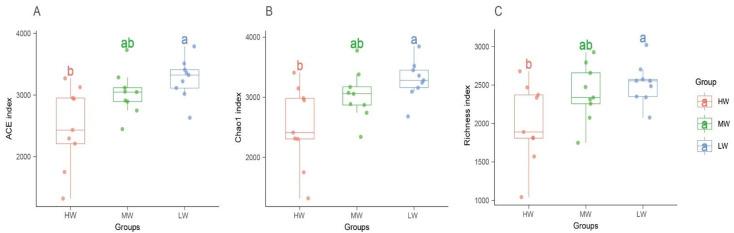
Diversity of ACE (**A**), chao1 (**B**), and richness index (**C**) of soil fungal communities under a natural moisture gradient on the south shore grassland ecosystem of Hulun Lake. HW: high water content; MW: medium water content; LW: low water content. Boxplots with different letters above the box indicate significantly different means (*p* < 0.05).

The fungal species diversity (ACE, chao1, and richness index) was significantly different under soil water contents. Additionally, there was an increasing trend with the decrease in SWC (Figure 3). However, the fungal community diversity (Shannon, Simpson, and InvSimpson’s index) had no significant difference under soil water contents and showed an increasing trend with the decrease in SWC. The community diversity was highest under the MW group (Figure S4).

### 3.4. Fungal Community Composition and Structure under a Natural Moisture Gradient

**Figure 4:**
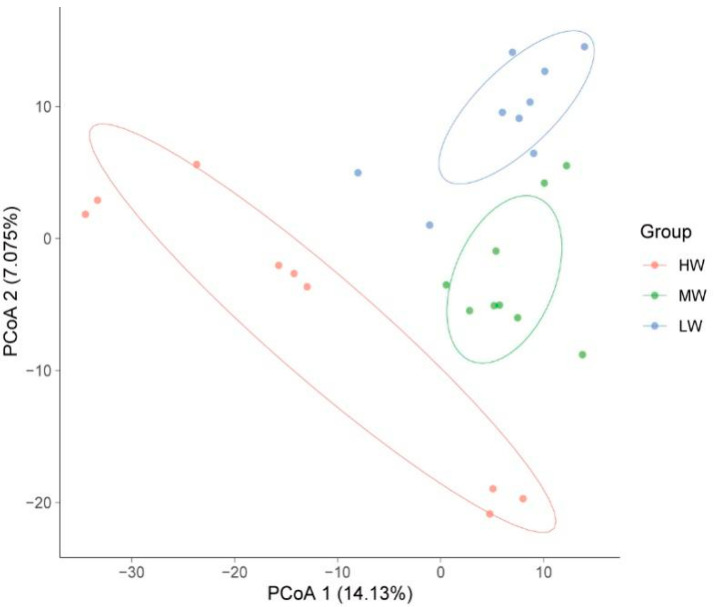
PCoA map of fungal communities under a natural moisture gradient on the south shore grassland ecosystem of Hulun Lake. The percentage of variation indicated on each axis corresponds to a fraction of the total variation explained by the prediction.

**Figure 5:**
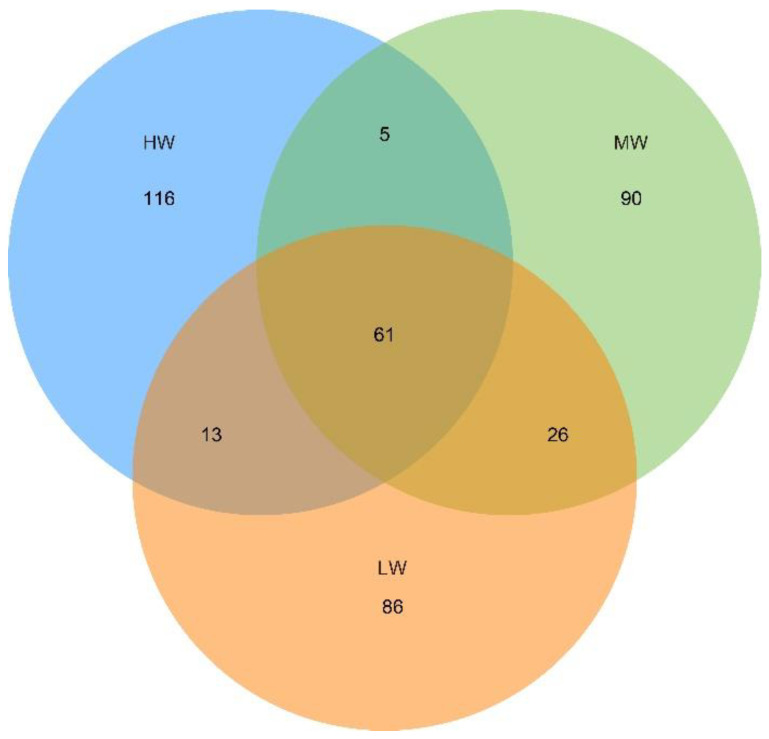
The unique ASVs and shared ASVs among the three groups are shown under a natural moisture gradient in the grassland ecosystem on the south shore of Hulun Lake, according to Venn diagrams. HW: high water content; MW: medium water content; LW: low water content.

Based on Euclidean distance, PcoA showed that the fungal communities were clearly separated under a natural moisture gradient, and the first and second axes explained 14.13% and 7.08%, respectively (Figure 4). HW, MW, and LW contained the shared ASV sequences of 61 soil fungi between and among them. The number of shared ASVs between LW and MW was higher, at 26. The next highest number of ASVs was shared between HW and LW, which was 13. Further, the number of ASVs shared between HW and MW was 5. The number of unique ASVs were 116, 90, and 86 under HW, MW, and LW, respectively, and then a total of 292 ASVs were affected by SWC in the soil (Figure 5).

### 3.5. Drivers of Fungal Community Composition

**Figure 6:**
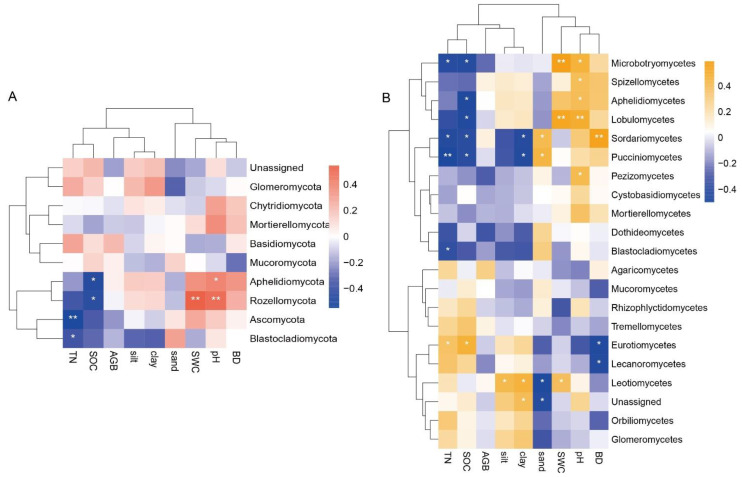
The relationship between the abundance of fungi phylum (**A**) or fungi class (**B**) and environmental factors. The relationship between environmental factors and the relative abundance of fungi was illustrated by Spearman’s correlation (* *p* < 0. 05; ** *p* < 0.01).

The fungal communities were significantly correlated with soil physicochemical properties (Figure 6). The dominant fungal phyla Blastocladiomycota, Ascomycota, Rozellomycota, and Aphelidiomycota were significantly negatively correlated with TN and SOC, and positively correlated with SWC and pH (Figure 6A). The dominant fungal phyla were most strongly correlated with SWC and pH. The trend was consistent with the dominant fungal class, and the dominant fungal class was mostly negatively correlated with TN and SOC and positively correlated with silt, clay, SWC, pH, and BD (Figure 6B).

## 4. Discussion

### 4.1. Environmental Factors under a Natural Moisture Gradient in Grassland Ecosystems on the South Shore of Hulun Lake

Soil fungi have improved stress and drought resistance for better adaptation to soil nutrient restriction and drought conditions [35]. Soil physicochemical properties and AGB were analyzed under a natural moisture gradient in the grassland ecosystem on the south shore of Hulun Lake. The results showed that there were significant differences in soil clay, TN, and SOC under the soil moisture gradient and showed a trend of increasing and then decreasing with the change in SWC from more to less (Table 2), supporting our first hypothesis. It has been pointed out that a moderate increase in SWC is beneficial to the growth of aboveground vegetation and also to the growth of soil microorganisms, and thus promotes the decomposition of soil humus and soil nutrient [35]. Moreover, moderate soil water content can improve soil texture and also increase the effectiveness of soil nutrients [36]. This is consistent with our finding that soil texture and soil nutrients are optimized when SWC is moderate.

### 4.2. Fungal Community Diversity under a Natural Moisture Gradient in Grassland Ecosystems on the South Shore of Hulun Lake

We established a phylogenetic tree by maximum likelihood method, and the fungi were divided into three main clades (phylum); with Ascomycota and Basidiomycota being the two largest clades (Figure 1). It waspreviously noted that the fungal phyla in Hulunbuir soils mainly consisted of Ascomycota and Basidiomycota [37], which is consistent with our findings (Table 3). It was pointed out that the main fungal groups of Hulunbeier soil were Ascomycota, Zygomycota, Chytridiomycota, and Basidiomycota, and about 98% of known fungal species belong to Ascomycota or Basidiomycota. In addition, Ascomycota and Basidiomycota had a faster evolutionary rate and more abundant species, so they were more suitable for survival under stress conditions [38]. The relative abundance of Ascomycota was higher in chronically flooded and non-flooded wetlands than that in indirectly flooded wetlands, suggesting that chronically watery and oxygen-deficient soil environments are not limiting the survival of all fungi under the Ascomycota phylum [39]. The relative abundance of the dominant phyla of soil fungi was not significantly different under different soil water contents (Table 3). However, the relative abundance of the major fungal classes Sordariomycetes and Dothideomycetes was higher in the HW group and significantly different under different SWCs (Table 3, Figure S3). Sordariomycetes and Dothideomycetes are relatively abundant in the phylum Ascomycota and are saprophytic fungi with a high tolerance, playing an important role in the carbon cycle as degraders of plant biomass in ecosystems [35,40]; this was a similar finding in our study. The evolutionary rates of these two fungi are different, and Sordariomycetes is the fastest and Dothideomycetes is the second fastest [38]. In this paper, the relative abundance of Sordariomycetes was higher than that of the Dothideomycetes (Table 4).

The species diversity (ACE, chao1, and richness indices) of the fungal communities showed an increasing trend from the near lake to far lake (Figure 3). Some studies have pointed out that soil fungi have good drought tolerance and can adapt to soil nutrient and water limitation in the ecosystem [35]. Fungi are able to grow under water-scarce conditions and can form spores to tolerate desiccation [41]. Therefore, when the SWC gradually decreases from the near lake to far lake, the environment is more suitable for the growth of fungal communities and increases their diversity. In addition, previous studies have pointed out that the long-term flooding state and long-term anthropogenic disturbance greatly change soil physicochemical conditions, and thus significantly decrease fungal biomass, abundance, and diversity, resulting in changes in soil microflora under a flooding state [42]. Therefore, we suggest that the higher SWC with the decreased oxygen content is unsuitable for the survival of aerobic soil fungi, resulting in a decrease in the diversity of the fungal community.

### 4.3. Fungal Community Composition under a Natural Moisture Gradient in Grassland Ecosystems on the South Shore of Hulun Lake

The natural moisture gradient in the grassland ecosystem on the south shore of Hulun Lake caused a significant variation in microbial taxonomic. Horton et al. [43] also observed the similar findings that hydrological environmental spatial differentiation contributed to the different correspondences of surface plants, leading to a large difference in microbial structure and function. Further, based on Euclidean distances, the fungal communities of the three treatments were clustered (Figure 4). However, the explanation rate was low, due to the establishment of a short moisture gradient strip. The study concluded that water fluctuations affect lake ecosystems, and can consequently affect wetland soils and soil microorganisms [44]. Additionally, organic elements in the soil are exchanged through water as a medium [45]. The overall soil environment changes, so the structure of the soil microbial community also changes [46].

Under HW, MW, and LW, 116, 90, and 86 unique ASVs, respectively, were observed in our study (Figure 5), showing that the moisture gradient significantly altered the soil microbial community composition. Previous studies have shown that the number of fungal Operational Taxonomic Units (OTUs) in non-waterlogged meadows is significantly higher than that in perennial waterlogged swamps [39]. In addition, it is believed that the number of soil fungal OTUs in non-flooded wetlands is not only higher than that in wetlands with long-term flooding status, but also higher than that in wetlands with intermittent flooding status [21]. In this paper, the number of unique fungal ASVs increased gradually with increasing SWC. However, the number of shared ASVs was higher in the low SWC environment. It showed that the unique 292 ASVs were affected more by SWC; and the shared ASVs were less affected by SWC (Figure 5). A higher frequency (and degree) of coexistence between microbial taxa in lower arid environments was observed by Wang et al. [47], suggesting that the higher precipitation can enhance microbe–microbe interactions. Thus, the SWC changes largely altered the composition of the soil fungal community, which was consistent with the results of existing studies [48].

### 4.4. Major Drivers of Fungal Community in Grassland Ecosystems on the South Shore of Hulun Lake

Soil physicochemical properties are important factors in the variation of fungal communities [49]. Soil nutrients are the main source of microbial metabolism and their own energy synthesis, and play an important role in the distribution of fungal communities [50]. Fungal communities can be positive [51], negative [39], or uncorrelated [52] with soil nutrients (SOC, TN, peroxidase). Moisture connectivity can weaken spatial distances, and thus to some extent, exhibits a delicate distance decay relationship in nearshore soils [53]. The relative importance of these drivers is conflicting, and changes in even small scale or environmental factors are important factors influencing the differences in fungal communities [54]. In this paper, we derived from Spearman’s correlation that Sordariomycetes had a significant negative correlation with soil nutrients (*p* < 0.05). The relative abundance of Sordariomycetes decreased when soil nutrient content was higher (Figure 6B). Further, it was noted that fungal diversity and soil nutrients jointly explained the variation in plant aboveground productivity, and fungal diversity and plant productivity were modulated by other environmental factors [55,56]. Wang et al. [57] found a significant positive relationship between fungal diversity and aboveground productivity after excluding the effects of other soil abiotic factors. In this paper, most of the fungal-dominant species were positively correlated with AGB, and the results were similar to the previous studies. However, this paper has not shown that the relationship between fungal-dominant species and AGB is jointly regulated by other environmental factors, so we can take this as an entry point for in-depth study in subsequent research. In addition, soil fine matter is an important factor for soil microorganisms, and soil texture affects the diversity and material composition of fungi [58]. In wetland ecosystems, because soil clay and sand content can protect microorganisms by forming a protective film, they also affect soil physicochemical properties [59]. A significant correlation between soil texture (clay and sand) and fungal classes was found in our study (Figure 6B). In the HW treatment, the increased content of soil clay led to higher relative abundance of the dominant classes Sordariomycetes and Dothideomycetes (Table 4). Secondly, SWC was the most important environmental factor in this paper, and Dothideomycetes, Sordariomycetes, and Microbotryomycetes were positively correlated with SWC (Figure 6B), indicating that the fungi relatively prefer environments with high SWC. In summary, SWC can affect soil fungal communities by influencing soil texture and nutrients and thus change soil fungal communities [21,60,61].

## 5. Conclusions

A natural moisture gradient in the grassland ecosystem on the south shore of Hulun Lake caused differences in the soil fungal community diversity and structural composition. SWC, as the most significant factor, altered the soil texture and nutrients, and thus affected the fungal communities. The fungal species diversity tended to decrease with increasing SWC. Furthermore, although the fungal β-diversity clustered significantly, their differences were not significant under different soil water contents. Ascomycota and Basidiomycota were the main fungal phyla when SWC was high. Additionally, Sordariomycetes and Dothideomycetes as the main dominant fungal classes had high relative abundance. Moreover, the unique ASVs of fungi were significantly affected by SWC and their numbers increased with increasing SWC. In general, the fungal community structural composition was stable in the high water environment, indicating that the fungal communities in the grassland ecosystem on the south shore of Hulun Lake are more likely to survive in the near-lake environment.

## Figures and Tables

**Table 1:** Locations of sampling sites on the south shore meadow ecosystem of Hulun Lake.

Group	Longitude (°E)	Latitude (°N)	Ellipsoid Height (m)
HW-1	117.2738889	48.58972222	481.29
HW-2	117.2872222	48.58166667	493.26
HW-3	117.2955556	48.57222222	509.89
MW-1	117.2855556	48.56194444	505.17
MW-2	117.2763889	48.54444444	511.63
MW-3	117.2680556	48.52027778	562.77
LW-1	117.2597222	48.49972222	545.77
LW-2	117.2541667	48.47055556	547.94
LW-3	117.2527778	48.465	544.57

**Table 2:** Soil physicochemical properties under a natural moisture gradient on the south shore grassland ecosystem of Hulun Lake. HW: high water content; MW: medium water content; LW: low water content. The values are mean ± standard error of mean (n = 9). Different letters indicate significant differences (*p* < 0.05) according to Duncan’s post-hoc test.

Environmental Factors	HW	MW	LW
Soil water content (%)	4.80 ± 1.18 a	2.17 ± 0.09 b	1.85 ± 0.14 b
Silt	0.05 ± 0.03 a	0.31 ± 0.46 a	0.04 ± 0.03 a
Clay	2.43 ± 0.70 b	4.08 ± 0.43 a	3.18 ± 0.25 ab
Sand	97.52 ± 0.72 a	95.61 ± 0.56 b	96.78 ± 0.27 ab
pH	7.63 ± 0.20 a	7.10 ± 0.11 a	7.35 ± 0.36 a
Bulk density (g cm^−3^)	1.27 ± 0.02 a	1.23 ± 0.02 a	1.27 ± 0.03 a
Total nitrogen (mg g^−1^)	0.51 ± 0.14 b	1.13 ± 0.13 a	0.96 ± 0.13 a
Soil organic carbon (mg g^−1^)	4.05 ± 1.20 b	8.16 ± 0.61 a	7.12 ± 0.72 a
Aboveground biomass (g m^−2^)	87.72 ± 17.72 a	122.01 ± 10.07 a	120.96 ± 4.86 a

**Table 3:** Relative abundances of fungal phyla under a natural moisture gradient on the south shore grassland ecosystem of Hulun Lake. The values are mean ± standard error of mean (n = 9). Different letters indicate significant differences (*p* < 0.05) according to Duncan’s post-hoc test. HW: high water content; MW: medium water content; LW: low water content.

Phylum	HW	MW	LW
Ascomycota	80.14 ± 3.46 a	67.76 ± 7.03 a	70.89 ± 3.78 a
Unassigned	13.22 ± 2.96 a	12.96 ± 2.50 a	13.91 ± 2.06 a
Basidiomycota	5.45 ± 1.7 a	16.07 ± 8.15 a	14.44 ± 4.73 a
Others	1.19 ± 0.38 a	3.19 ± 1.33 a	0.75 ± 0.09 a

**Table 4:** Relative abundances of fungal classes under a natural moisture gradient on the south shore grassland ecosystem of Hulun Lake. The values are mean ± standard error of mean (n = 9). Different letters indicate significant differences (*p* < 0.05) according to Duncan’s post-hoc test. HW: high water content; MW: medium water content; LW: low water content.

Class	HW	MW	LW
Sordariomycetes	33.01 ± 5.48 a	28.24 ± 3.31 a	25.03 ± 2.80 a
Unassigned	19.56 ± 3.73 a	17.90 ± 2.48 a	20.90 ± 2.82 a
Dothideomycetes	26.32 ± 3.97 a	14.07 ± 2.93 b	20.87 ± 3.18 ab
Agaricomycetes	4.71 ± 1.77 a	15.97 ± 8.16 a	14.39 ± 4.74 a
Eurotiomycetes	6.29 ± 2.36 a	11.98 ± 2.34 a	10.83 ± 3.93 a
Leotiomycetes	4.14 ± 1.73 a	7.24 ± 4.81 a	4.55 ± 2.11 a
Pezizomycetes	3.32 ± 1.51 a	0.66 ± 0.32 a	1.84 ± 1.26 a
Others	2.62 ± 0.76 a	3.94 ± 0.30 a	1.60 ± 0.30 a

## Data Availability

The data presented in this study are available on request from the corresponding author. The data are not publicly available due to that the data requires multiple authors’ agreement for usage.

## Supplementary Materials

The following supporting information can be downloaded at: https://www.mdpi.com/article/10.3390/jof9050549/s1, Figure S1: Sample site clustering analysis complete connection. Figure S2: The fungal rarefaction curves curve under a natural moisture gradient. HW: high water content; MW: medium water content; LW: low water content. Figure S3: The relative abundance of fungal phylum (A) and class (B) under soil moisture gradient on the south shore grassland ecosystem of Hulun Lake. HW: high water content; MW: medium water content; LW: low water content. Different color lines represent different species. Figure S4: Diversity of Shannon (A), Simpson (B), and InvSimpson’s index (C) of soil fungal communities under soil moisture gradient on the south shore grassland ecosystem of Hulun Lake. HW: high water content; MW: medium water content; LW: low water content. Boxplots with different letters above the box indicate significantly different means (*p* < 0.05).
