# Supplementary material for: A Natural Moisture Gradient Affects Soil Fungal Communities on the South Shore of Hulun Lake, Inner Mongolia, China
Source: J Fungi (Basel). 2023 May 10;9(5):549. doi: 10.3390/jof9050549 (PMC10218956; doi:10.3390/jof9050549)
Supplement: Supplementary file 1 [file jof-09-00549-s001.zip › jof-2263596-supplementary.pdf]

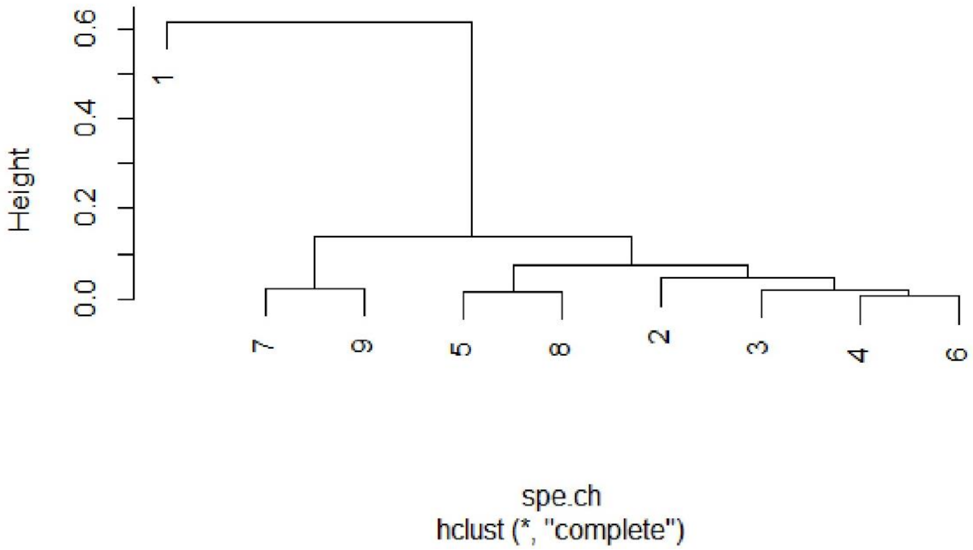

Figure S1. Sample site clustering analysis-complete connection.

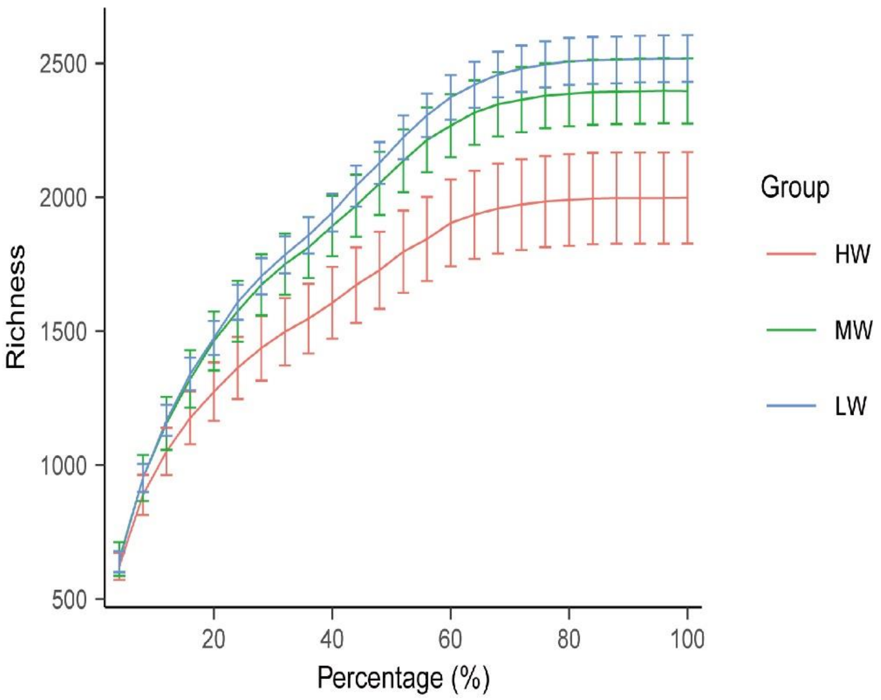

Figure S2. The fungal rarefaction curves curve under a natural moisture gradient. HW: high water content; MW: medium water content; LW: low water content.

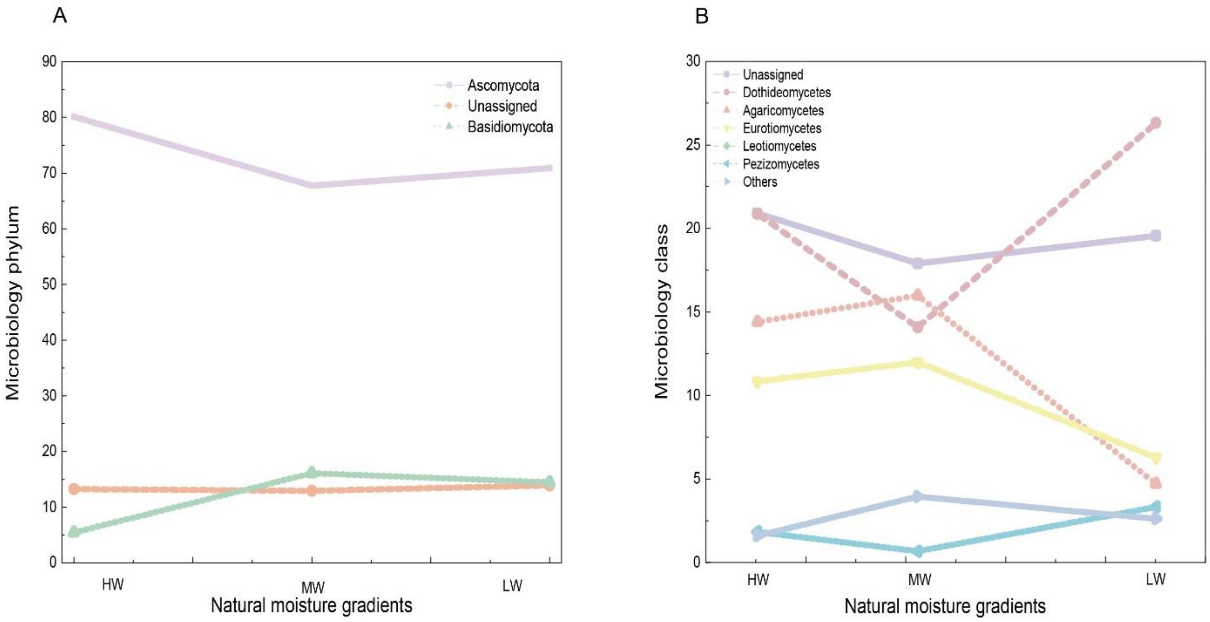

Figure S3. The relative abundance of fungal phylum (A) and class (B) under soil moisture gradient on the south shore grassland ecosystem of Hulun Lake. HW: high water content; MW: medium water content; LW: low water content. Different color lines represent different species.

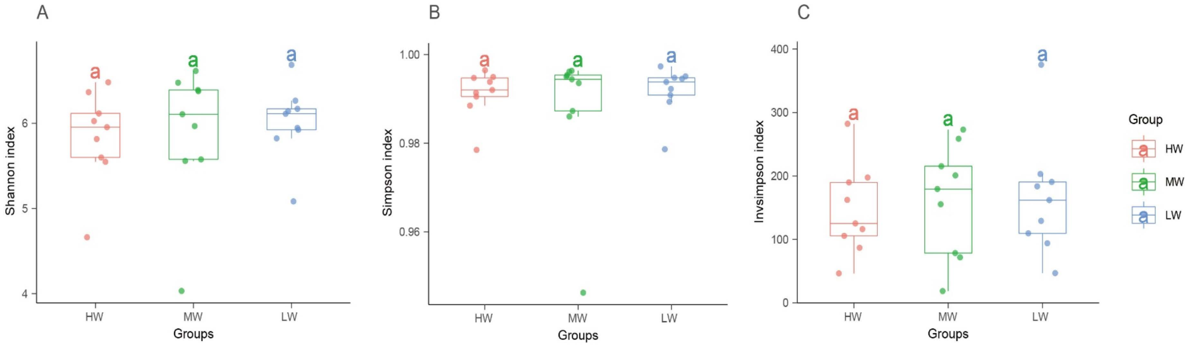

Figure S4. Diversity of Shannon (A), Simpson (B) and Invsimpson's index (C) of soil fungal communities under soil moisture gradient on the south shore grassland ecosystem of Hulun Lake. HW: high water content; MW: medium water content; LW: low water content. Boxplots with different letters above the box indicate significantly different means ($P < 0.05$).
